# From Warm to Cold: Feeding Cold Milk to Preterm Infants with Uncoordinated Oral Feeding Patterns

**DOI:** 10.3390/nu17091457

**Published:** 2025-04-26

**Authors:** Louisa Ferrara-Gonzalez, Ranjith Kamity, Zeyar Htun, Vikramaditya Dumpa, Shahidul Islam, Nazeeh Hanna

**Affiliations:** 1 Division of Neonatology, Department of Pediatrics, NYU Langone Hospital—Long Island, NYU Grossman Long Island School of Medicine, Mineola, NY 11501, USA; 2 Division of Neonatology, Department of Pediatrics, Arkansas Children’s Hospital, University of Arkansas for Medical Sciences, Little Rock, AR 72205, USA; 3 Biostatistics Unit, Office of Academic Affairs, Northwell Health, New Hyde Park, NY 11042, USA

**Keywords:** dysphagia, cold milk, oral feeding, preterm, safety, neonate, swallow, coordination

## Abstract

**Background/Objectives**: Premature infants frequently experience feeding difficulties due to the disrupted coordination of sucking, swallowing, and breathing, increasing the risk of airway compromise. In adults with dysphagia, cold liquids can enhance swallowing by stimulating sensory receptors in the pharyngeal mucosa. We previously demonstrated that short-duration feeding with cold liquid significantly reduces dysphagia in preterm infants; however, the impact of an entire feeding with cold milk remains unexplored. This study aimed to evaluate the safety of cold milk feedings in preterm infants with uncoordinated feeding patterns and their impact on their feeding performance. **Methods**: Preterm infants with uncoordinated feeding patterns (*n* = 26) were randomized to be fed milk at either room temperature (RT) or cold temperature (CT) using an experimental, randomized crossover design. We monitored axillary and gastric content temperatures, mesenteric blood flow, and feeding performance. **Results**: There were no significant differences in mesenteric blood flow Doppler measurements or axillary body temperatures between the CT and RT feeding conditions. However, a reduction in gastric content temperatures of 3.6 °F and 2.7 °F was observed at one and thirty minutes following CT feeding, respectively. No evidence of cold stress, increased episodes of apnea or bradycardia, gastric residuals, or emesis was noted in infants during or after the CT feeding condition. Feeding performance outcomes did not differ significantly regarding milk transfer rate (*p* = 0.781) or proficiency (*p* = 0.425). However, the quality score on the Infant-Driven Feeding Scale (IDFS) showed a significant improvement following CT feeding (*p* = 0.001). **Conclusions**: Cold milk feeding can be a safe therapeutic option for preterm infants. This underscores the potential for further comprehensive investigations to evaluate cold milk feeding as an effective therapeutic strategy for managing feeding and swallowing difficulties in preterm infants. The study was registered at clinicaltrials.org under #NCT04421482.

## 1. Introduction

Preterm infants exhibit a developmental trajectory in oral motor skills from 30 to 45 weeks post-menstrual age [1]. This progression begins with a limited coordination of sucking, swallowing, and breathing, eventually leading to a fully integrated sucking pattern. However, during this developmental phase, certain preterm infants may continue to display signs of neurological and physiological immaturity, which can adversely impact their success with oral feeding [1,2,3]. An uncoordinated feeding pattern is characterized by a lack of rhythmicity in the total sucking activity and or an inability to synchronize sucking, swallowing, and breathing [4,5]. This can place some preterm infants at an increased risk for dysphagia, characterized by aspiration or laryngeal penetration, resulting in complications such as pneumonia, respiratory disease, or poor growth [6,7,8]. Approximately 30% of very low-birth-weight preterm infants will be diagnosed with dysphagia [9,10,11,12]. Given this prevalence, appropriate evaluation and treatment of dysphagia are of clinical importance to improve the medical outcomes of these at-risk preterm infants.

Conventional therapies for preterm infants with dysphagia include slow-flow nipples, elevated side-lying positioning, pacing sucking bursts, and feed thickening, though these approaches may have limited efficacy [13,14,15,16,17,18,19,20,21,22]. Thickened feeds stimulate pharyngeal mechanoreceptors to enhance swallowing but are less effective with breast milk due to the enzymatic breakdown of thickeners [19,20,21,22,23]. Few prior studies have specifically addressed formula or milk ingestion temperature, and those available have primarily focused on short-term physiological outcomes such as gastric emptying [24,25]. Feeding cold milk has emerged as a potential adjunct therapy. It activates pharyngeal thermo-receptors, enhancing sensory input and triggering more effective swallowing. In adults, cold liquids have shown benefits in reducing aspiration through similar mechanisms involving cranial nerve-mediated sensory pathways [26,27,28]. Our group demonstrated a reduction in aspiration rates from 71% to 26% with short-duration cold barium feeding in dysphagic preterm infants while under videofluoroscopy [29].

While previous studies demonstrated the safe use of cold feedings [24,25,30,31,32], concerns remain regarding potential digestive dysfunction [33], cold stress [34,35,36], and adverse physiological effects such as respiratory distress, desaturation, delayed gastric motility, increased residuals, and emesis [35,36]. Preterm infants are also more susceptible to NEC and hypothermia, which are associated with poor outcomes [35]. It is unclear whether cold milk affects mesenteric blood flow or feeding performance in this population. This study aims to evaluate whether a full cold milk feeding induces cold stress, alters mesenteric blood flow, or negatively impacts feeding performance in preterm infants with uncoordinated feeding. We hypothesize that cold milk will be well-tolerated, without significant physiological or feeding-related adverse effects.

## 2. Materials and Methods

### 2.1. Participants

NYU Langone’s institutional review board approved this study and informed written consent was obtained before enrollment. The study was performed in accordance with the Declaration of Helsinki and is not intended to report on long-term outcomes. The study was registered at clinicaltrials.org #NCT04421482. All participants received care in the Neonatal Intensive Care Unit (NICU) at NYU Langone Hospital—Long Island. Eligible participants were preterm infants (<37 weeks gestational age at birth) with a post-menstrual age (PMA) of 34–42 weeks at the time of enrollment and a documented uncoordinated feeding pattern, identified using the Quality Score from the Infant-Driven Feeding Scales (IDFS) [37], which is routinely administered by nursing and medical staff in our unit. All enrolled infants were stable and maintained normothermia in an open crib. Infants were excluded if they had intrauterine growth restriction or a history of gastrointestinal pathology, including necrotizing enterocolitis, gastroschisis, malabsorption, diarrhea, or omphalocele. Infants with neonatal abstinence syndrome were also excluded due to potential neurobehavioral disturbances (e.g., irritability, tremors, excessive sucking, and high-pitched crying) that could affect feeding behavior [38]. There were no exclusions based on gender, race, or ethnicity.

Participants’ demographic information collected included gender, race, gestational age at birth, birth weight, PMA, chronological age at the time of the study enrollment, and clinical information, such as feeding milestones and medical diagnoses.

### 2.2. Methods

Each participant was exposed to two oral feeding conditions—room temperature (RT, 68–77 °F) and cold temperature (CT, 39–48 °F)—in a randomized crossover design. The order of feeding (start with RT or CT) was determined by a coin flip at the bedside, and both feeds were conducted within a 12 h period to minimize potential confounding factors, while also allowing for ample time to negate any residual effects of the cold stimuli [24,25,39]. This approach was selected for its practicality in this crossover design, where each infant served as their own control, thus minimizing inter-individual variability and reducing the risk of selection bias. Although allocation concealment was not employed, the short 12 h interval between feeding conditions further limited the potential for systematic bias.

In the RT condition (control), infants received their usual milk (formula or breast milk) at room temperature, while in the CT condition (intervention), the same milk was provided at cold temperature. Feeding was performed by a trained research team member with infants positioned in an elevated side-lying posture and using a slow-flow nipple [40,41]. An infant-driven approach to feeding was used consistently for all participants, with pauses introduced (pacing) when signs of stress or intolerance appeared, allowing the infant time to breathe or suck on an empty nipple.

Several procedural controls were implemented to ensure consistency across all feeding conditions and participants. For the CT condition, milk was stored in a refrigerator until it reached approximately 39–48 °F. For the RT condition, milk was left at the bedside for no longer than two hours before the feeding assessment. Prior to each feeding, milk temperature was measured using a liquid thermometer (Red Lantern TP3001 Digital Thermometer, Tianjin, China), which was sanitized after each use according to institutional protocols.

#### 2.2.1. Safety Assessment

To ensure infant safety and monitor for potential signs of cold stress, several measures were implemented throughout the study. Axillary temperatures were taken immediately before and after each feeding using a digital thermometer. For infants with a nasogastric tube (NGT) in place, gastric content temperatures were also measured; this step was omitted in infants without an NGT to avoid unnecessary invasive procedures.

Prior to each feeding session, NGT placement was confirmed via auscultation with a neonatal stethoscope to ensure accurate positioning in the stomach. Once verified, feeding commenced, and data on feeding performance were collected. Immediately following each feeding, 2 mL of gastric content was aspirated using a 3 mL syringe. The temperature of the aspirate was measured using a digital thermometer, with the syringe held at its base to reduce heat transfer from the handler’s hands. In the RT condition, the gastric content temperature served as the baseline reference. In the CT condition, gastric content temperature was measured at 1, 10, and 30 min post-feeding to monitor the return to baseline. Additionally, clinical signs of cold stress—including tachypnea, desaturation, apnea, bradycardia, increased gastric residuals, and emesis—were observed and documented during and after each feeding session.

Doppler ultrasound (Sonosite Edge Ultrasound System, Sonosite, Inc., Bothell, WA, USA) was used to assess superior mesenteric artery (SMA) blood flow as a marker of intestinal perfusion. Peak systolic velocity (PSV) and end-diastolic velocity (EDV) were measured directly, and the resistive index (RI) was calculated using the formula RI = (PSV − EDV)/PSV [42,43].

Each participant underwent six Doppler ultrasound assessments: once one hour before each feeding session (baseline), and again at 30 and 60 min after each feeding. All measurements were conducted at the bedside with the infant in a supine position. To ensure reliability and consistency, each measurement was performed twice and by the same operator.

#### 2.2.2. Feeding Performance Assessment

We adopted objective and subjective methods of measuring feeding performance to evaluate the infants’ feeding skills during both feeding conditions. These measurements were captured and documented at 5 min intervals throughout the feeding. The 5 min intervals commenced at the moment the infant latched onto the nipple and began their first sucking burst. From the initiation of sucking, the timer was set to run continuously for 5 min, regardless of any pauses in sucking by the infant. The only instance where the timer was stopped before reaching the 5 min mark was if the infant demonstrated clear signs of physiological instability. This 5 min interval method was selected to improve the precision of subjective assessments. At each interval, the feeding was briefly paused to allow the research team to record observations on the data collection sheet.

The objective measure of the Oral Feeding Skills score (OFS) was administered during both feeding conditions, RT and CT [44]. We collected the following data: (a) the percentage of the volume consumed within the initial five minutes, (b) the total volume that was prescribed, (c) the overall volume ingested throughout the entire feeding session, and (d) the total duration of active feeding. Using these metrics, the OFS score was calculated as described by Lau and Smith, providing values for proficiency (percentage of volume taken at 5 min relative to volume prescribed) and rate of transfer (mL/min) [44].

The subjective assessment of suck–swallow–breathe coordination was conducted using the Quality Score from the IDFS, which rates infants’ coordination on an ordinal scale from 1 to 5, with 1 indicating the highest level of coordination and 5 the lowest [37]. Infants were scored within each 5 min interval based on the scale’s established criteria. The IDFS is recognized as a valid and reliable tool for supporting the safe and effective development of oral feeding skills in preterm infants and for guiding evidence-based interventions [45]. Bedside nurses and the trained research team member who performed the feedings completed a 5 h universal online module training, as well as a 21-question post-education questionnaire to ensure understanding.

Two additional subjective measures—Stress Cues and Need for Pacing—were also evaluated at each 5 min interval using ordinal scales ranging from 0 to 3. These scoring systems, developed for the purposes of this study (see Table 1), include the Stress Scale, which is based on Als’ Synactive Theory and identifies cues from the motoric, behavioral, and attention/interaction subsystems, and the Pacing Scale, supported by prior research [16,17,46,47]. Furthermore, any cardiorespiratory events—including apnea, bradycardia, or oxygen desaturation—were monitored and recorded using the Life Scope TR monitor (Nihon Kohden America, Foothill Ranch, CA, USA) [48].

### 2.3. Statistical Analyses

Demographic characteristics were summarized using median (interquartile range) for continuous variables and frequency (percentage) for categorical variables, as appropriate. Normality of continuous variables was assessed using the Kolmogorov–Smirnov test, histograms, and Q-Q plots. For each clinical outcome, changes between the RT and CT conditions were calculated and presented as mean differences with corresponding 95% confidence intervals. Paired sample t-tests were used for normally distributed data, while the Wilcoxon signed-rank test was applied for non-normally distributed variables.

For ultrasound measurements, changes from baseline were calculated at 30 and 60 min post-feeding for both RT and CT conditions. The mean differences in these changes were also reported with 95% confidence intervals. All statistical analyses were performed using SAS 9.4 (SAS Institute Inc., Cary, NC, USA), with a *p*-value < 0.05 considered statistically significant.

## 3. Results

A total of 26 preterm infants were enrolled in the study, including 5 infants who underwent pre- and post-feeding Doppler ultrasound assessments to evaluate mesenteric blood flow. As presented in Table 2, the median gestational age at birth was 31 weeks and 6 days. At the time of the study, the median day of life was 28 days, and the median PMA was 36 weeks and 1 day. All participants had prior experience with oral feeding, with a median of 8 days of partial oral feeding before enrollment. The randomization process assigned the RT feeding as the initial feeding for 14 infants, while 12 infants received the CT feeding first.

To assess mesenteric blood flow via Doppler ultrasound, we selected infants born at <30 weeks gestation due to their higher risk for necrotizing enterocolitis (NEC). Table 3 provides demographic details for the five infants who underwent these Doppler evaluations.

### 3.1. Safety Assessment

As presented in Table 4, there was no significant reduction in participants’ axillary body temperature following CT feeding compared to RT feeding (*p* = 0.618). Gastric temperature measurements were performed only in infants who already had a nasogastric tube (NGT) in place at the time of the study (*n* = 7), to avoid introducing an invasive procedure solely for research purposes. By one minute after feeding, the temperature of the cold milk had increased from its initial range of 39–48 °F to an average of 83.3 °F upon reaching the stomach. The gastric content temperature following CT feeding was only 3.6 °F lower than that observed after RT feeding at the one-minute time point. Despite the relatively small difference, this reduction was statistically significant (Table 4). Importantly, no signs of cold stress were observed in any of the infants who received cold milk, indicating good overall tolerance to the intervention.

As shown in Table 5, there were no significant differences in mesenteric blood flow Doppler findings between CT and RT feedings. In both conditions, peak systolic velocity (PSV) and end-diastolic velocity (EDV) increased at 30 min post-feeding, with a mean rise of 13.5 cm/sec and 10.7 cm/sec, respectively, compared to pre-feeding baseline values. At 60 min post-feeding, both PSV and EDV decreased from their 30 min values but remained elevated relative to baseline, with a mean increase of 5.2 cm/sec and 5.1 cm/sec, respectively. Resistive index (RI) values remained unchanged at both 30 and 60 min compared to pre-feeding measurements.

Given that the initial five cases showed no impact of cold milk on mesenteric blood flow, further Doppler assessments were not performed to avoid unnecessary procedures for additional participants.

### 3.2. Feeding Performance Assessment

A total of 21 infants completed both pre- and post-feeding performance assessments. As shown in Table 6, there were no statistically significant differences in OFS levels between the two feeding conditions (*p* = 0.734). Additionally, objective measures—including proficiency (% volume taken at 5 min relative to volume prescribed), rate of transfer (mL/min), and overall volume transferred—did not differ significantly between RT and CT conditions.

Similarly, subjective assessments of stress cues and the need for pacing showed no significant differences between RT and CT conditions. However, coordination of sucking, swallowing, and breathing, as measured by the Infant Dysphagia Feeding Scale (IDFS), demonstrated a statistically significant improvement with CT feeding (*p* = 0.001). The mean IDFS Quality Score was 3.2 (SD 0.7) during the RT condition and 2.6 (SD 0.9) during the CT condition, indicating better coordination during CT feedings (Table 6). To assess clinical relevance, individual score changes were analyzed: 10 out of 21 infants demonstrated a 1–3 point improvement, 10 showed no change, and only 1 infant had a 1-point decline. These findings suggest that, beyond statistical significance, CT feeding may support individualized feeding strategies in preterm infants.

## 4. Discussion

This pilot study demonstrates that a full single CT feeding is well tolerated in preterm infants, with no observed clinical adverse events. There were no significant changes in axillary temperature or mesenteric blood flow parameters following CT feedings compared to RT feedings. Additionally, CT feeding did not negatively affect feeding performance. Notably, CT feeding was associated with improved coordination of sucking, swallowing, and breathing in preterm infants with uncoordinated feeding patterns. This was evidenced by a statistically significant improvement in the IDFS Quality Score, with mean scores improving from 3.2 during RT feedings to 2.6 during CT feedings (*p* = 0.001). To explore the clinical significance of the findings, 10 of 21 infants showed a 1–3 point improvement in IDFS scores, 10 remained unchanged, and 1 declined by 1 point. To assess the clinical relevance of changes in IDFS scores, we calculated Cohen’s *d* effect size using the mean difference divided by the pooled standard deviation of the two groups [49]. An effect size of 0.74 was obtained, indicating a moderate to large effect. These findings suggest that CT feeding may offer meaningful clinical insights to guide individualized feeding strategies in preterm infants. To the best of our knowledge, this is one of the few studies to assess the safety of cold milk feeding in preterm infants, and the only study to evaluate mesenteric blood flow responses in this population following CT feeding.

Although the average gastric content temperature during CT milk feeding was statistically lower than during RT feeding, this small difference does not appear clinically significant. No changes in mesenteric blood flow or signs of feeding intolerance were observed following CT feeding. Bissinger et al. reported that the optimal ambient temperature for maintaining thermal stability in very low birth weight (VLBW) infants ranges from 71 °F to 80 °F [34]. While their study focused on external cold exposure, our investigation of internal cold exposure through CT milk feeding yielded consistent findings. The average gastric temperature post-CT feeding was 82.9 °F—higher than the optimal ambient range—and did not result in cold stress. This suggests that internal exposure to cold via milk feeding, similar to external exposure, does not adversely affect thermal stability in VLBW infants [33].

For preterm infants, the standard practice involves administering gavage feedings of human milk or formula at room temperature (68–77 °F) until oral feeding is established. This suggests that milk introduced into the stomach at ambient temperature is generally well-tolerated without gastrointestinal complications. In our study, cold milk administered orally reached the stomach at approximately 83.3 °F, higher than the typical room temperature range used for direct gastric feedings via nasogastric or gastric tubes. Although our study did not investigate the effects of delivering cold milk directly into the stomach via NGT, our findings are consistent with those from a study in adult volunteers, which demonstrated that gastric content temperatures normalized to within 1 °F of core body temperature within 30 min after ingesting a cold beverage [50]. Importantly, no gastrointestinal adverse events—such as vomiting, feeding intolerance, increased gastric residuals, or changes in stool frequency or consistency—were observed in our participants following CT feeding. These results are also in line with CDC guidance, which confirms that infants can safely consume cold milk [51].

One potential concern with cold milk feeding is its theoretical risk of inducing mesenteric vasoconstriction and altering intestinal blood flow. However, our findings indicate that the impact of CT feeding on mesenteric perfusion in preterm infants is minimal. No significant changes were observed in Superior Mesenteric Artery blood flow, as reflected by stable resistive index measurements taken before, and at 30 and 60 min after CT feeding, compared to RT feeding, further supporting the safety of CT feeding.

Our previous research has demonstrated the efficacy of using cold milk for a short duration (limited to 5 swallows) in reducing penetration and aspiration in premature infants with dysphagia [29]. While using cold milk may lower the risk of penetration and aspiration, this does not automatically imply an improvement in overall feeding efficiency or the effectiveness of transferring milk. While the primary objective of this study was to assess the safety of administering a full feeding of CT milk to preterm infants, we also explored its impact on feeding performance. Our findings indicate that CT feeding did not negatively affect feeding performance. In fact, some infants showed improvement in coordination of sucking, swallowing, and breathing when fed CT milk compared to RT milk. This potential enhancement in coordination is consistent with adult studies suggesting that cold liquids increase sensory input by stimulating thermal receptors in the oral cavity and hypopharynx, which may enhance motor coordination [26,27,28]. However, these findings must be interpreted in the context of the infants’ clinical status. At the time of the study, the median PMA was 36 weeks and 1 day, with a median of 8 days of oral feeding experience, and infants were consuming approximately 50% of their feeding volume orally. Thus, while CT feeding may support coordination in some cases, further research is needed before recommending it solely to enhance feeding performance. Nonetheless, for infants at risk of penetration or aspiration, CT milk appears to be a safe option without adverse impact on feeding performance.

While no significant differences were observed in the objective OFS measures, a notable improvement was detected in the subjective IDFS scores. This discrepancy likely reflects differences in the focus and sensitivity of the two tools. While both tools have strong sensitivity [52], they vary in clinical perspective. The OFS emphasizes quantitative outcomes such as volume intake and transfer rate [53], whereas the IDFS captures qualitative aspects of feeding coordination and infant-driven cues [16,37,54]. The IDFS may, therefore, be more sensitive to subtle improvements in feeding quality that are not reflected in volume-based metrics. These findings highlight the importance of using both objective and subjective assessments to obtain a more comprehensive evaluation of feeding performance in preterm infants. CT feeding had minimal effect on OFS but significantly improved IDFS scores. Some infants demonstrated strong OFS performance—ingesting larger volumes at faster rates—yet received lower IDFS scores due to poor coordination of sucking, swallowing, and breathing. This inverse relationship has been reported in previous studies [3,14,54,55,56] and reinforces the value of a co-regulated, cue-based, and infant-driven approach to feeding. Emerging evidence supports the benefits of this approach, particularly in preterm infants born at ≥28 weeks, showing accelerated feeding progression and reduced hospital length of stay [57,58,59]. Furthermore, infant-driven feeding has been associated with lower hospital readmission rates in the first year of life, improved long-term feeding outcomes, and reduced reliance on feeding therapy in the NICU [60,61]. These outcomes are attributed to feedings that prioritize physiologic stability, motor organization, and coordinated suck–swallow–breathe patterns, fostering a more positive and developmentally supportive feeding experience [1,3,53,54,60,62,63].

A limitation of this pilot study is the small sample size; however, significant findings were observed despite including only 26 participants. The limited number of subjects, particularly for the mesenteric blood flow assessments, increases the risk of a Type II error (false negative), which may limit the statistical power and generalizability of the findings.

Future studies with larger samples, utilizing computer-generated randomization and concealed allocation, extended intervals between CT and RT feeds or parallel-group designs are warranted to confirm our findings. Furthermore, this study evaluated only the short-term safety of a single cold milk feeding. Long-term effects on nutrient absorption, gastrointestinal health, and feeding development remain unknown and warrant future investigation. Energy expenditure and oxygenation status were not assessed in this study. Their inclusion in future research will provide a more comprehensive evaluation of the physiological impact of cold milk feeding. Another limitation was that blinding was not feasible due to the visible difference in milk temperature and the need for real-time safety monitoring by the same trained evaluators. This may introduce observer bias, especially in subjective assessments like the IDFS. Future studies should incorporate video recordings and blinded evaluations to enhance objectivity. While our study suggests that cold milk feeding may be safe, the application of cold milk feeding should be individualized based on gestational age, post-menstrual age, feeding readiness, thickened feeds and co-existing clinical factors. Tools like videofluoroscopic swallow studies (VFSS) may further guide individualized care in infants with more complex feeding needs [64].

While the primary objective was to evaluate the safety of CT feeding, further studies are needed to assess the efficacy of repeated CT feedings as a therapeutic intervention for neonatal dysphagia within a structured feeding protocol. Future research should also explore the combined use of CT milk with other strategies, such as feed thickening, to enhance therapeutic options for managing dysphagia in preterm infants.

## 5. Conclusions

Our findings indicate that a single cold milk feeding was safely tolerated in preterm infants, with no clinically significant changes in axillary or gastric temperature or mesenteric blood flow compared to RT feedings. Additionally, CT feeding did not negatively impact feeding performance in infants with uncoordinated feeding patterns.

These results support the potential for further research to evaluate the efficacy of ongoing CT feedings as a therapeutic strategy for feeding and swallowing difficulties in preterm infants. Given the favorable safety profile demonstrated in this study, CT feeding may be considered a viable option for infants with dysphagia when other interventions have been assessed. However, its use should be individualized, carefully assessed, and closely monitored to ensure safety and effectiveness for each infant.

## Figures and Tables

**Table 1:** Scoring rubric for subjective scales.

	0	1	2	3
Stress Scale	No behavioral stress cues observed	1–3 behavioral stress cues observed	4–10 behavioral stress cues observed	>10 behavioral stress cues observed
Pacing Scale	No pacing is offered; infant self-regulated suck–swallow–breathe coordination	Pacing is offered 1–3 times	Pacing is offered 4–10 times	Pacing is offered >10 times

**Table 2:** Demographics and clinical characteristics of infants who received pre- and post-feeding safety measures and performance measures (*n* = 26).

Variable	Frequency (Percentage) or Median (IQR)
Female sex, *n* (%)	15 (57.7%)
Gestational age at birth, median, weeks/days	31 6/7 (28 2/7, 32 4/7)
Birth weight, median, grams	1625 (837, 1966)
Day of life at the start of the study, median	28 (16, 73)
PMA (feeding introduction), median, weeks/days	34 5/7 (34, 36 6/7)
PMA at the start of the study, median, weeks/days	36 1/7 (35 1/7, 38 5/7)
Days of oral feeds at the start of the study, median	8 (5, 13)
Percent oral feeds at the start of the study, median	50 (40, 70)

**Table 3:** Demographics and clinical characteristics of infants who underwent ultrasound assessments (*n* = 5).

Variable	Frequency (Percentage) or Median (IQR)
Female sex, *n* (%)	3 (50%)
Gestational age at birth, median, weeks/days	26 5/7 (25, 29 2/7)
Birth weight, median, grams	615 (600, 635)
Day of life at the start of the study, median	27.5 (16, 73)
PMA (feeding introduction), median, weeks/days	37 2/7 (36 4/7, 37 5/7)
PMA at the start of the study, median, weeks/days	39 2/7 (38 5/7, 39 5/7)
Days of oral feeds at the start of the study, median	10 (2, 14)
Percent oral feeds at the start of the study, median	27 (7, 30)

**Table 4:** Safety and outcome comparisons between RT and CT (*n* = 21).

Variable	RT Mean (SD)	CT Mean (SD)	Change (RT-CT) 95% CI	*p*-Value
Body Temp (°F) Pre-feeding	98.3 (0.3)	98.3 (0.2)	−0.1 (−0.2, 0.1)	0.379
Body Temp (°F) Post-feeding	98.2 (0.3)	98.3 (0.3)	0 (−0.2, 0.1)	0.618
Gastric Temp (°F) 1 min Post-feeding §	86.7 (0.4)	83.3 (1.1)	3.6 (2.0, 5.2)	**<0.001**
Gastric Temp (°F) 10 min Post-feeding §	86.7 (0.4)	82.9 (1.1)	4.0 (2.3, 5.4)	**<0.001**
Gastric Temp (°F) 30 min Post-feeding §	86.7 (0.4)	84.2 (1.0)	2.7 (1.3, 4.1)	**0.003**

*p*-values are from paired *t*-test for normally distributed variables and Wilcoxon signed rank test for non-normally distributed variables. SD—standard deviation, F—Fahrenheit, §—effective sample size, *n* = 10, bold indicates a significant *p*-value.

**Table 5:** Mesenteric blood flow Doppler findings (*n* = 5).

Variable	Change from Baseline RT Mean (SD)	Change from Baseline CT Mean (SD)	CT-RT Difference Mean (95% CI)
Changes at 30 min			
PSV	13.5 (8.4)	10.7 (3.8)	−0.1 (−11, 10.7)
EDV	3.5 (3.4)	0.8 (2.8)	−1.9 (−7.2, 3.3)
RI	0 (0)	0 (0)	0 (0, 0.1)
Changes at 60 min			
PSV	5.2 (5)	5.1 (9.7)	1.1 (−10, 12.3)
EDV	0.4 (1.1)	−0.1 (0.6)	−0.5 (−3.1, 2.1)
RI	0 (0)	0 (0)	0 (0, 0.1)

Table shows ultrasound measures obtained for superior mesenteric artery blood flow parameters: peak systolic velocity (PSV) and end diastolic velocity (EDV) were measured, and resistive indices (RI) were calculated. Measurements were obtained before feeding, after 30 min and after 60 min of feeding for both room temperature (RT) and cold temperature (CT) feedings for each subject. Peak systolic velocity (PSV), end diastolic velocity (EDV), resistive index (RI), room temperature feeding (RT), cold temperature feeding (CT), standard deviation (SD), 95% confidence interval (95% CI).

**Table 6:** Feeding performance outcome comparisons between RT and CT (*n* = 21).

Variable	RT Mean (SD)	CT Mean (SD)	Change (RT-CT) 95% CI	*p*-Value
Total length of feeding (min)	12.4 (4.6)	14.3 (4.6)	−1.9 (−4.8, 1.1)	0.203
Total volume ingested for feeding (mL)	25.6 (16.7)	28 (14.4)	−2.4 (−6.6, 1.7)	0.238
OFS Level Ɨ	2.3 (1.3)	2.2 (1.2)	0.1 (−0.4, 0.6)	0.734
Proficiency	23.5 (12.9)	25.5 (15.3)	−2 (−7, 3)	0.425
Rate of Transfer (mL/min)	2 (1.2)	2 (1)	0 (−0.3, 0.4)	0.781
Overall Volume Transfer (mL) Ɨ	54 (35.8)	58.7 (31.3)	−4.7 (−13.5, 4.1)	0.147
IDF Quality Raw Score Ɨ	3.2 (0.7)	2.6 (0.9)	0.6 (0.2, 1)	**0.001**
Pacing Scale Ɨ	1.4 (1)	1 (0.9)	0.4 (0, 0.8)	0.057
As. Bs. Ds. Ɨ	0.3 (0.9)	0.2 (0.7)	0.1 (−0.1, 0.4)	0.375
Stress Scale Ɨ	1.6 (0.6)	1.7 (0.8)	0 (−0.3, 0.3)	0.933

*p*-values are from paired *t*-test for normally distributed variables and Wilcoxon signed rank test for non-normally distributed variables; bold indicates a significant *p*-value; SD, standard deviation; Ɨ, not normally distributed; RT, room temperature; CT, cold temperature; OFS, oral feeding skills score; As. Bs. Ds., apneas, bradycardias, desaturations. Proficiency: percentage of volume taken at 5 min relative to the volume prescribed.

## Data Availability

The datasets generated from the data analysis are not available publicly due to patient privacy restrictions and regulations. A limited, de-identified dataset may be available from the corresponding author upon reasonable request.

## Abbreviations

The following abbreviations are used in this manuscript:

CT	Cold temperature
RT	Room temperature
IDFS	Infant-Driven Feeding Scale
NICU	Neonatal Intensive Care Unit
PMA	Post-menstrual age
NGT	Nasogastric tube
SMA	Superior mesenteric artery
PSV	Peak systolic velocity
EDV	End diastolic velocity
RI	Resistive index
OFS	Oral feeding skills
NEC	Necrotizing enterocolitis
VLBW	Very low birth weight
